# Flowerlike CeO_2_ microspheres coated with Sr_2_Fe_1.5_Mo_0.5_O_x_ nanoparticles for an advanced fuel cell

**DOI:** 10.1038/srep11946

**Published:** 2015-07-08

**Authors:** Yanyan Liu, Yongfu Tang, Zhaohui Ma, Manish Singh, Yunjuan He, Wenjing Dong, Chunwen Sun, Bin Zhu

**Affiliations:** 1 Hubei Collaborative Innovation Center for Advanced Organic Chemical Materials, Faculty of Physics and Electronic Science, Hubei University, Wuhan, Hubei 430062; 2 Hebei Key Laboratory of Applied Chemistry, College of Environmental and Chemical Engineering, Yanshan University, 438 Hebei Street, Qinhuangdao, 066004, P.R. China; 3 Key Laboratory for Renewable Energy, Beijing Key Laboratory for New Energy Materials and Devices, Beijing National Laboratory for Condensed Matter Physics, Institute of Physics, Chinese Academy of Sciences, Beijing 100190, China; 4 Department of Energy Technology, Royal Institute of Technology, Stockholm, SE-10044, Sweden; 5 Beijing Institute of Nanoenergy and Nanosystems, Chinese Academy of Sciences, Beijing 100083, China

## Abstract

Flowerlike CeO_2_ coated with Sr_2_Fe_1.5_Mo_0.5_O_x_ (Sr-Fe-Mo-oxide) nanoparticles exhibits enhanced conductivity at low temperatures (300–600 ^o^C), e.g. 0.12 S cm^−1^ at 600 ^o^C, this is comparable to pure ceria (0.1 S cm^−1^ at 800 ^o^C). Advanced single layer fuel cell was constructed using the flowerlike CeO_2_/Sr-Fe-Mo-oxide layer attached to a Ni-foam layer coated with the conducting transition metal oxide. Such fuel cell has yielded a peak power density of 802 mWcm^−2^ at 550 ^o^C. The mechanism of enhanced conductivity and cell performance were analyzed. These results provide a promising strategy for developing advanced low-temperature SOFCs.

Low temperature solid oxide fuel cells (LT-SOFCs) are highly desired as advanced energy conversion and storage devices, in terms of the improvement on issues of mismatch of thermal expansion coefficient of various components and rapid degradation of the components of cells operating at high temperature^1^,^2^. Also LT-SOFCs, say below 600 ^o^C, have higher Nernst voltages, which offer a feasible strategy to obtain excellent cell performance^3^. However, the electrolyte with sufficiently high conductivity and electrocatalyst function are big challenges for development of high performance LT-SOFCs.

Ceria is an important oxygen ion conducting and catalysis material. It has attracted intense attention due to its property to improve the oxygen reduction reaction process through reversely reducing Ce^4+^ to Ce^3+^, high oxygen storage capacity as well as good oxide ion conductivity of 10^−1^ S cm^−1^ at 800 ^o^C. These properties are comparable to the conductivity of yttrium stabilized zirconia (YSZ) at 1000 ^o^C^4^,^5^. Flowerlike textured ceria (F-CeO_2_) was firstly synthesized and used as an internal catalytic layer for hydrocarbon fueled SOFCs to yield a peak power density of 654 mW cm^−2^ at operating temperature 600 ^o^C by Sun *et al.*^6^,^7^,^8^. As reported in the literature, this unique open mesoporous structure has great potential kinetic advantages because of its high surface area and improved dispersion of the active secondary components.

In this work, considering high active surface and large porous surface properties of the flowerlike CeO_2_, we further modify the surface of F-CeO_2_ using semiconducting Sr-Fe-Mo-oxide since Sr_2_Fe_1.5_Mo_0.5_O_x_ (SFM) has high catalytic activity, stable redox property and excellent electrical conductivity^9^,^10^,^11^. The electronic carriers are generated from the mixed coexistence of redox couples Fe^2+^/Fe^3+^ and Mo^6+^/Mo^5+^, which enhances the ionic conduction due to the introduction of oxygen vacancies into the lattice^12^,^13^,^14^. Liu *et al.* studied overall electrical properties as regards excellent redox stability^9^. As reported, the conductivities of SFM reached to 310 S cm^−1^ and 550 S cm^−1^ at 780 ^o^C in hydrogen and air atmospheres, respectively^1^,^9^. Particularly at low temperatures, SFM also displayed excellent redox stability and high electrical conductivity in both air (8–60 S cm^−1^) and hydrogen (4–8 S cm^−1^) environments at 400–600 ^o^C^9^. These properties indicate that SFM is excellent for both anode and cathode at LT-SOFCS and also at intermediate temperature.

Recently, Dong *et al.* developed single layer fuel cell (SLFC) using the SFM as one component in a composite with SDC-Na_2_CO_3_. They made the SLFC using the 30 wt.% SFM and 70 wt.% SDC-Na_2_CO_3_, which exhibits the highest OCV (open circuit voltage) of 1.05 V and output of 360 mW cm^−2^ at 750 ^o^C^1^. This also proved that the SFM had good redox electrocatalyst function to make the SLFC work. They also pointed out that to achieve the high performance SLFCs, the electron conductivity (41 S cm^−1^ in their synthesized SFM) had to be balanced by ions (0.05 S cm^−1^ level) for their SDC-Na_2_CO_3_^1^. In our SLFCs, SFM was mainly utilized as the semiconducting material in F-CeO_2_/SFM-oxide composite to obtain balanced electronic and ionic conductivities. Through coating electronic conducting SFM on the ionic conducting ceria, we obtained a novel functional semiconductor-ion composite for high performance at low temperature, bellow 600 ^o^C, SLFCs. In recent years, semiconducting and ionic materials have been discovered with the new functionality which can effectively promote the fuel cell redox and ion transport processes at particle levels^15^,^16^. This unique property has enabled novel single layer fuel cells (SLFCs) possible, which provides many chances to improve the performance of fuel cells without the limitation of electrolytes^17^,^18^. This fuel cell technology was selected as a research breakthrough in SOFCs field, which was named ‘Three in One’ by Nature Nanotechnology^19^. The SLFCs differ from the conventional three-component configuration of anode/electrolyte/cathode in solid oxide fuel cells in both the technology and science^20^.

## Results

### Morphology and structural characterization of F-CeO_2_ and F-CeO_2_/Sr-Fe-Mo-oxide composite

**Figure 1:**
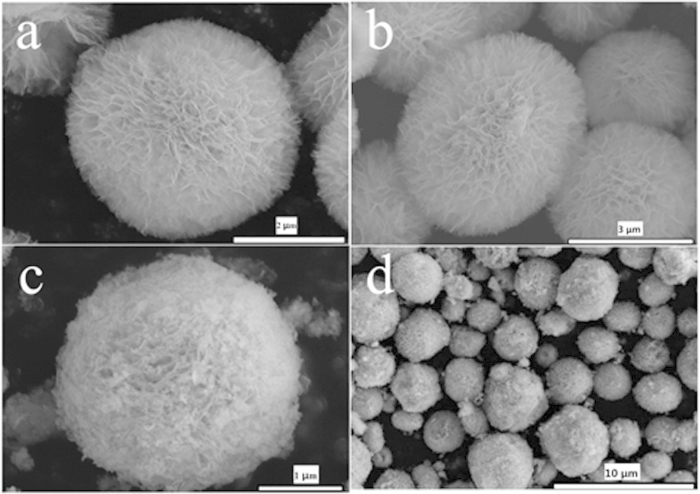
Representative SEM images of (**a**,**b**) flowerlike CeO_2_ and (**c**,**d**) CeO_2_ coated with Sr-Fe-Mo-oxide.

**Figure 2:**
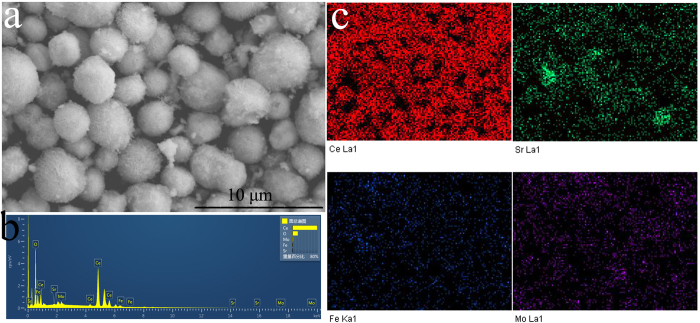
(**a**) SEM image; (**b**) EDX and (**c**) Elemental mapping of the flowerlike ceria coated with Sr-Fe-Mo-oxide after calcination at 750 ^o^C for 2 h.

Figure 1 shows the mesoporous ceria microspheres with a flowerlike texture observed by a field-emission scanning electron microscope (FE-SEM). The flowerlike CeO_2_ particles (see Fig. 1a,b) show an open three-dimensional porous and hollow microsphere composed of numerous interweaved thin flakes as the petals. These microspheres are nearly monodisperse with diameter approximately 2 to 5 μm. Fig.1c,d give an overview of the F-CeO_2_/Sr-Fe-Mo-oxide composite, which show Sr-Fe-Mo-oxide particles are highly dispersed on the surface of flowerlike CeO_2_ microspheres without any structural change. These Sr-Fe-Mo-oxide particles were coated on the surface of ceria microsphere. Due to the mesoporous structure of the flowerlike ceria microspheres, it provides sufficient spaces to integrate with sheet-like Sr-Fe-Mo-oxide particles. Sr-Fe-Mo-oxide, as a high electronic conducting material, is coated on the surface of ionic conductor ceria, to make a semiconducting-ionic conducting composite material for the single layer fuel cell. To further analyze the composition of the composite layer, the energy dispersive x-ray (EDX) analysis and elemental mapping of the surrounding flowerlike ceria particles were shown in Fig. 2. The results confirm the existences of Sr, Fe, and Mo, indicating that Sr-Fe-Mo-oxide particles were successfully loaded onto the flowerlike ceria microspheres. It can be seen from the elemental mapping images (Fig. 2c) that various elements of Sr-Fe-Mo-oxide uniformly distributed on the surface of ceria to form F-CeO_2_/Sr-Fe-Mo-oxide composite type material.

**Figure 3:**
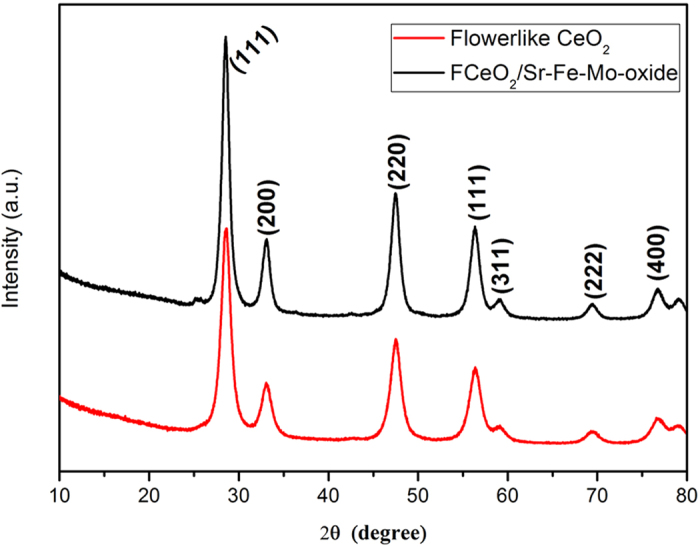
XRD patterns of (**a**) flowerlike CeO_2_ and (**b**) CeO_2_ coated with Sr-Fe-Mo-oxide.

The phases and purity of as-prepared flowerlike ceria and F-CeO_2_/Sr-Fe-Mo-oxide samples were examined by x-ray diffraction (XRD) patterns. All diffraction peaks in the patterns of Fig. 3a can be ascribed to a face-centered cubic fluorite structure CeO_2_ (JCPDS 34–0394)^21^,^22^. It is worthy noted that all the diffraction peaks of flowerlike CeO_2_ coated with Sr-Fe-Mo-oxide composite have no shift compared with that of pure ceria. This indicates that the Sr-Fe-Mo-oxide in ceria did not induce any detectable structural changes and doping effect. Hence, our material coating approach is successful. The grain size of F-CeO_2_ is approximately 6 nm, estimated from the strongest (111) peak with Scherrer equation. Further detailed phase analysis is limited since no peaks of Sr-Fe-Mo-oxide could be detected by XRD. This may be due to the coating limitation at a composition of 4.0 mol% which may be under the XRD detection level. Anyhow, the uniform coating of Sr-Fe-Mo-oxide particles has succeeded in unique F-CeO_2_/Sr-Fe-Mo-oxide semiconductor-ionic composite material with high electrical and electrocatalyst properties.

### Electrical properties

**Figure 4:**
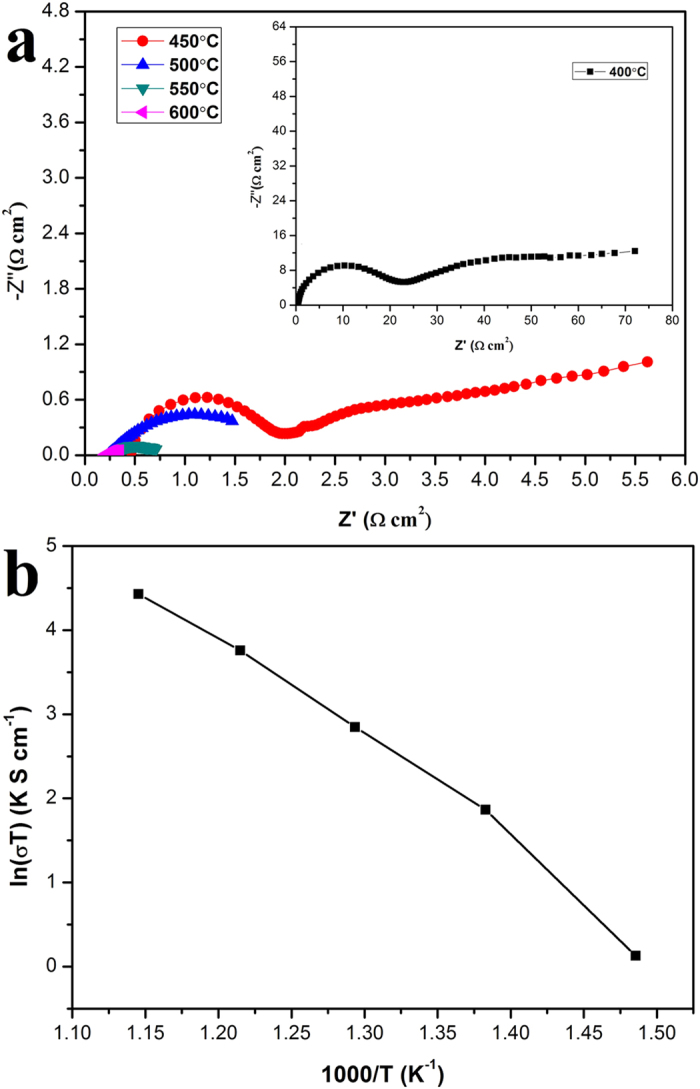
(**a**) Electrochemical impedance spectra (EIS) at open-circuit voltage (OCV) of fuel cell measured at various temperature from 600 ^o^C to 400 ^o^C upon cooling at an interval of 50 ^o^C and (**b**) Arrhenius plots corresponding to Nyquist curves using the as-prepared F-CeO_2_/Sr-Fe-Mo-oxide composite as a single component fuel cell.

Figure 4 displays the typical Nyquist plots for a F-CeO_2_/Sr-Fe-Mo-oxide SLFC obtained by EIS measurements, between 400 and 600 ^o^C. In general, an impedance spectrum of solid state materials exhibits successive semicircles in the complex plane, including the bulk, grain boundary and electrode polarization processes^23^,^24^. At low temperatures, e.g. 400 ^o^C and 450 ^o^C, three arcs can be observed. Two located at higher frequency correspond to the grain interior and grain boundary while the start appearing tail in the low frequency is associated with the interface of F-CeO_2_/Sr-Fe-Mo-oxide and NCAL, *i.e.* electrode polarization process. The semicircles, representing the grain interior and interface polarization, disappeared with the increase of temperature. The Arrhenius plots obtained from the corresponding Nyquist curves are shown in Fig. 4b.

### Fuel cell performances

**Figure 5:**
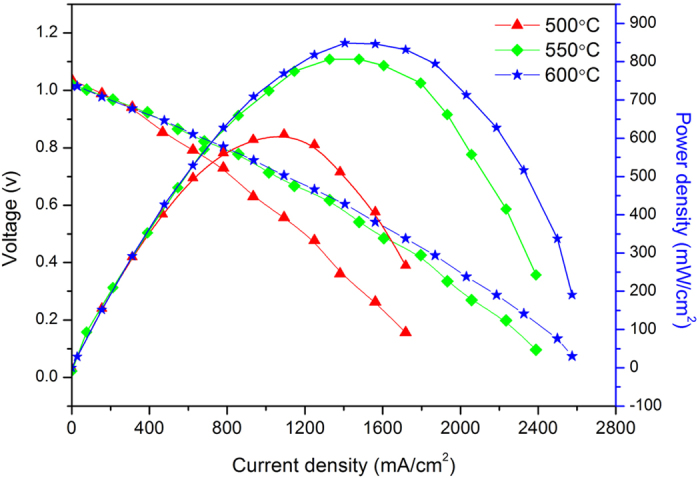
Electrochemical performance of fuel cell based on as-prepared CeO_2_/SFM-oxide material with a cell configuration: Ni pasted NCAL | F-CeO_2_/Sr-Fe-Mo-oxide | NCAL pasted on Ni foam measured at various operating temperature of 500 ^o^C, 550 ^o^C and 600 ^o^C.

**Figure 6:**
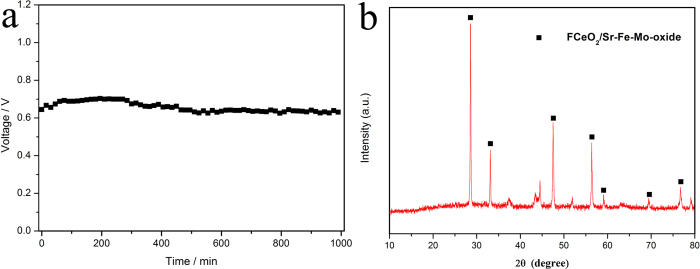
(**a**) The operation stability test result of SLFC at a current density of 312.5 mA/cm^2^ at 530 ^o^C; b) XRD patterns of F-CeO_2_/Sr-Fe-Mo-oxide layer after long-term stability test.

Figure 5 shows I-V and I-P characteristics for F-CeO_2_/Sr-Fe-Mo-oxide fuel cells, measured at the operating temperature of 500 ^o^C, 550 ^o^C and 600 ^o^C, respectively. It shows that all obtained open circuit voltage (OCV) of the fuel cells above 1.0 V at various temperatures, as a prerequisite for obtaining excellent performance, indicating that these as-prepared materials have superior catalytic activity as a single layer material. It can be seen that the maximum current densities were approximately 1718, 2389, and 2574 mA cm^−2^ at 500 ^o^C, 550 ^o^C and 600 ^o^C, respectively. The peak output power density is 610 mW cm^−2^ at 500 ^o^C, and increases to 802 mW cm^−2^ at 550 ^o^C. At 600 ^o^C, a peak power density reaches about 848 mW cm^−2^, which is consistent with the results of electrical conductivity measured with AC impedance spectrum. These results demonstrate that the conductivity of F-CeO_2_/Sr-Fe-Mo-oxide composite is sufficiently high. We further evaluated the operation stability of the F-CeO_2_/Sr-Fe-Mo-oxide fuel cell. The device was operated at a current density of 312.5 mA cm^−2^ at 530 ^o^C for over 16 h. The voltage change was recorded over time during operation. It can be seen from Fig. 6a that the device has a relatively good durability with the minimal voltage degradation. Generally, the main factor for degradation can be attributed to the increased polarization resistance^25^. The cell voltage slightly degrades at around 5 h. This could be resulted from the change of cerium valence state in the F-CeO_2_/Sr-Fe-Mo-oxide composite in the initial stage^26^,^27^. Some Ce^4+^ ions were reduced to Ce^3+^ ions at H_2_ input side^28^,^29^,^30^ leading to the coexistence of both cerium ions valence states. The phase analysis of the F-CeO_2_/Sr-Fe-Mo-oxide layer after long-term stability test was displayed in Fig. 6b. The same peak positions to the F-CeO_2_/Sr-Fe-Mo-oxide composite can be clearly identified in the XRD patterns. This suggests that the material structure has no changes. Some additional peaks were identified because the NCAL layer was mixed into the material when the sample was scraped from the tested device pellet. Further longer life test is limited by our testing device. After the durability test, we found some rusting surface on the steel chamber of the testing device used, which caused a slight degradation due to the testing device resistance growing with the measurement.

**Figure 7:**
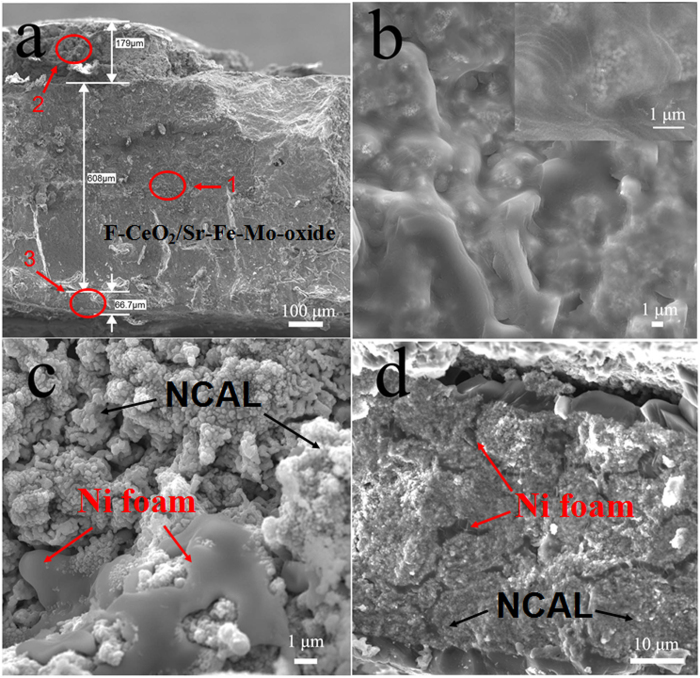
SEM micrographs of cross-sectional images of **a**) fuel cell configuration; **b**) F-CeO_2_/Sr-Fe-Mo-oxide layer; **c**) Ni foam pasted NCAL in cathode side; **d**) Ni foam pasted NCAL in anode side after stability test.

Figure 7 exhibits the SEM micrographs of the device cross-sectional images for details after long-term stability test. As shown in Fig. 7a, the F-CeO_2_/Sr-Fe-Mo-oxide single layer is 0.6 mm approximately. The thickness of Ni-foam pasted NCAL layer in cathode side and anode side is 179 and 66 μm, respectively. Fig. 7b,c and d are the enlargement for the area 1, 2 and 3 in Fig. 7a. It can be seen that the flowerlike texture of ceria has been destroyed in high temperature and long-term test. However, the initial flowerlike structure results in hierarchical flakes, which induces the Sr-Fe-Mo-oxide homogenously coated on the special flowerlike morphology and microstructure. This may benefit greatly the F-CeO_2_/Sr-Fe-Mo for high performance LT-SOFCs. The composition of the three regions analyzed by EDX are displayed in Table 1. It can be seen that oxygen content significantly reduced due to the reduction of NCAL-oxide in reducing atmosphere. As shown in Fig. 7c,d, the integration of NCAL and Ni foam layer in cathode and anode sides can contribute to the current collection. Simultaneously, the Ni-foam porous structure provides the tunnels for gas transfer to reach at the F-CeO_2_/Sr-Fe-Mo-oxide layer.

## Discussion

**Figure 8:**
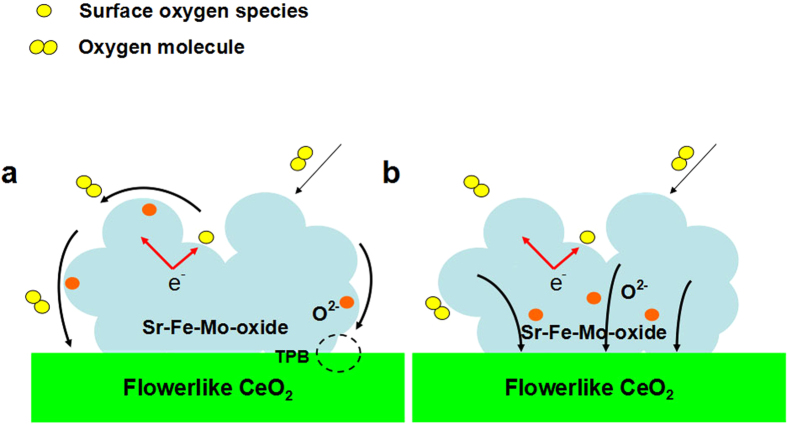
Schematically illustration of (**a**) Surface path; (**b**) bulk path of ORR process.

Sr-Fe-Mo-oxide, as a state-of-art mixed ionic and electronic conducting material with a good catalytic activity was successfully coated on the F-CeO_2_ particles, which provides two paths to promote oxygen reduction reaction (ORR) process occurring at the cathode side of SOFC during operation: bulk and surface (interface) oxygen transfer and transport into F-CeO_2_^31^,^32^, as shown schematically in Fig. 8a,b.

(1) In the surface process, the Sr-Fe-Mo-oxide particles as an active second phase was highly dispersed on the surface of the F-CeO_2_ microspheres. The transport of oxygen ions through the electrode was enhanced by the F-CeO_2_ particles due to the formation of the highly active Sr-Fe-Mo-oxide sites and continuous oxygen ion conducting network on the surface of the F-CeO_2_ microspheres. The process can be demonstrated clearly in Fig. 8a. Ceria with a fluorite structure is an oxide ionic conductor and its conductivity can be further enhanced by controlling the preparation atmosphere^33^. In this case, this flowerlike texture provides more surface area to integrate the Sr-Fe-Mo-oxide particles, resulting in large extension of the Sr-Fe-Mo-oxide/F-CeO_2_/gas triple phase boundary (TPB) length to increase the oxygen surface exchange and charge transfer^34^,^35^,^36^,^37^. The enhancement of oxygen surface exchange reaction leads to improved cell performance^16^. The F-CeO_2_ surface kinetic process is increased via decreasing the re-equilibration time when the Sr-Fe-Mo-oxide particles are deposited^38^. Usually, the kinetic processes dominate the interfacial polarization resistance during the operation of fuel cells^39^. Consequently, the resistance is greatly reduced due to the increase of surface kinetic processes. Thus, the cell performance is increased.

(2) In the bulk process, Sr-Fe-Mo-oxide particles coated on the flowerlike CeO_2_ microsphere surface can promote ORR process occurring at the cathode side through the following mechanism: Sr-Fe-Mo-oxide has high oxygen mobility and large vacancies to interact with the oxygen molecules absorbed on the Sr-Fe-Mo-oxide particle surfaces, which provide fast oxygen dissociation and transport paths for the oxygen and oxygen ions via Sr-Fe-Mo-oxide bulk and surfaces into the F-CeO_2_ particles to complete the ORR process (as shown in Fig. 8b). Therefore, the two-path promoted ORR process and conductivity enhancement have been achieved by coating Sr-Fe-Mo-oxide on the F-CeO_2_ microspheres.

In summary, we developed a novel semiconducting and ionic material with new functionalities by coating Sr-Fe-Mo-oxide particles on the flowerlike CeO_2_ microsphers, which can significantly promote the fuel cell oxygen reduction reaction and ion transport processes. Based on an advanced single layer fuel cell technology, a peak power density of 802 mW cm^−2^ was obtained using hydrogen as a fuel and air as an oxidant operated at 550 ^o^C. This work may lead to a new path to develop advanced LT-SOFCs.

## Methods

### Materials preparation

The flowerlike F-CeO_2_/Sr-Fe-Mo-oxide composite sample was prepared by the following two steps. Firstly, the flowerlike CeO_2_ was prepared by a hydrothermal method and subsequent cacination as reported in the literature^8^. Secondly, the preparation of flowerlike F-CeO_2_/Sr-Fe-Mo-oxide composite sample was as follows. Strontium nitrate, iron nitrate and ammonium molybdate were mixed according to the stoichiometric ratio of 2:1.5:0.5. The mixture was ground thoroughly in an agate mortar to obtain homogeneous Sr_2_Fe_1.5_Mo_0.5_Ox precursor. Then the as-preparation flowerlike ceria and Sr_2_Fe_1.5_Mo_0.5_Ox precursor were grinded again with the content of 4 mol% Sr_2_Fe_1.5_Mo_0.5_O_x_ precursor to obtain homogeneous flowerlike CeO_2_-Sr_2_Fe_1.5_Mo_0.5_O_x_ precursor. The resulting materials were transferred into ceramic crucible following a sintering process at 750 ^o^C for 2 h. The final sample obtained via thorough grinding was noted as F-CeO_2_/Sr-Fe-Mo-oxide composite.

The NCAL is initials for Ni_0.8_Co_0.15_Al_0.05_Li-oxide was purchased from Tianjin Baomo Joint Hi-Tech venture, China.

### Characterization of materials

Powder X-ray diffraction (XRD) analysis was carried out using D-max-2500 X-ray diffractometer (Rigaku Corp., Japan) with Ni-filtered Cu Kα radiation (λ = 1.54056 Å). The patterns were recorded at the 2θ range of 10–90^o^ with step size of 0.02^o^. Scanning electron microscopy (SEM) was performed on a cold field emission scanning electron microscope (Hitachi S-4800).

Electrochemical impedance spectroscopy (EIS) was carried out to measure the electrical conductivity of the prepared material F-CeO_2_ coated with Sr-Fe-Mo-oxide composite from 600 ^o^C to 400 ^o^C upon cooling at an interval of 50 ^o^C. The measured frequency was ranging from 0.01 Hz to 1 MHz under a bias voltage of 10 mV.

### Fuel cell preparation and measurement

The fuel cell was fabricated using a new fuel cell technology in a simple symmetrical configuration using the prepared F-CeO_2_/Sr-Fe-Mo-oxide sample as the single component material and NCAL was pasted on foam nickel as anode side and cathode side. Simultaneously, the conducting transition metal oxide NCAL can play a role as current collector. The two more pure foam nickel were pressed slightly on both sides while mount the pellet on the testing device to provide the channels to exhaust water vapor. The cell configuration can be simply denoted as Ni pasted NCAL NCAL | F-CeO_2_ | Sr-Fe-Mo-oxide | NCAL pasted on Ni foam. The fuel cell were compressed at a pressure of 200 MPa for 2 min with an active area of 0.64 cm^2,^ the thickness and diameter were 2 mm and 13 mm, respectively.The cell was tested on the computerized instrument (IT8511 + 120 V/30A/150 W) using hydrogen as fuel and air as oxidant operated at 550^o^C, respectively. The flow rate of H_2_ is in the range of 80 ~ 120 ml min^−1^ and the flow rate of air is 100 ml min^−1^under an atmospheric pressure.

## Additional Information

**How to cite this article**: Liu, Y. *et al.* Flowerlike CeO_2_ microspheres coated with Sr_2_Fe_1.5_Mo_0.5_O_x_ nanoparticles for an advanced fuel cell. *Sci. Rep.* **5**, 11946; doi: 10.1038/srep11946 (2015).

## Figures and Tables

**Table 1:** Composition for F-CeO_2_/Sr-Fe-Mo-oxide layer, Ni foam pasted NCAL in cathode side and anode side analyzed by EDX.

Regions	O/wt%	Fe/wt%	Sr/wt%	Mo/wt%	Ce/wt%	Co/wt%	Al/wt%	Ni/wt%
1 F-CeO_2_/Sr-Fe-Mo-oxide layer	26.14	0.31	0.63	0.58	72.34	−	−	−
2 Ni foam pasted NCAL in cathode side	10.80	−	−	−	−	15.57	1.67	71.95
3 Ni foam pasted NCAL in anode side	1.39	−	−	−	−	11.18	0.01	87.42
